# Order-to-Disorder Transition and Hydrogen Bonding in the Jahn–Teller Active NH_4_CrF_3_ Fluoroperovskite

**DOI:** 10.1021/acs.inorgchem.4c00931

**Published:** 2024-05-24

**Authors:** Øystein S. Fjellvåg, Bruno Gonano, Fabian L. M. Bernal, Salah B. Amedi, Jike Lyu, Vladimir Pomjakushin, Marisa Medarde, Dmitry Chernyshov, Kenneth Marshall, Martin Valldor, Helmer Fjellvåg, Bjørn C. Hauback

**Affiliations:** † Department for Hydrogen Technology, Institute for Energy Technology, P.O. Box 40, Kjeller NO-2027, Norway; ‡ Laboratory for Neutron Scattering and Imaging, Paul Scherrer Institute, Villigen-PSI CH-5232, Switzerland; § Chemistry Department and Center for Material Science and Nanotechnology, University of Oslo, Oslo NO-0315, Norway; ∥ Division for Research, Dissemination and Education, IT-department, University of Oslo, Oslo 0316, Norway; ⊥ Laboratory for Multiscale Materials Experiments, Paul Scherrer Institut, Villigen-PSI CH-5232, Switzerland; # Swiss-Norwegian Beam Lines at European Synchrotron Radiation Facility, 71 Avenue des Martyrs, Grenoble 38043, France

## Abstract

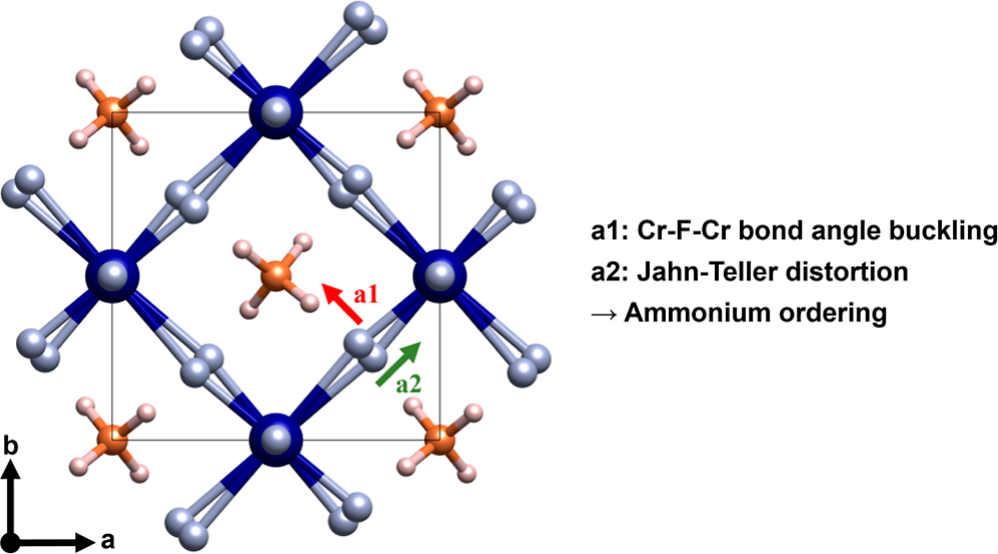

Large quantities of high-purity NH_4_CrF_3_ have been synthesized using a wet-chemical method, and its structural chemistry and magnetic properties are investigated in detail for the first time. NH_4_CrF_3_ is a tetragonal fluoroperovskite that displays an ordering of the ammonium (NH_4_^+^) groups at room temperature and C-type orbital ordering. The ammonium groups order and display distinct signs of hydrogen bonds to nearby fluoride anions by buckling the Cr–F–Cr angle away from 180°. The ammonium ordering remains up to 405 K, much higher than in other ammonium fluoroperovskites, indicating a correlation between the flexibility of the Jahn–Teller ion, the hydrogen bond formation, and the ammonium ordering. At 405 K, an order-to-disorder transition occurs, where the ammonium groups disorder, corresponding to a transition to higher symmetry. This is accompanied by a contraction of the unit cell from breaking hydrogen bonds, similar to the phenomenon observed in water ice melting. The compound orders antiferromagnetically with a Neél temperature of 60 K, an effective paramagnetic moment of 4.3 μ_B_, and a Weiss temperature of −33 K. An A-type antiferromagnetic structure is identified by neutron diffraction, with an ordered moment of 3.72(2) μ_B_.

## Abstract 2

NH_4_CrF_3_ is a Jahn–Teller distorted perovskite with ordered ammonium groups at room temperature. The ammonium groups order and display distinct signs of hydrogen bonds to nearby fluoride anions by buckling the Cr–F–Cr angle away from 180°. The complexity of the structural chemistry of NH_4_CrF_3_ results from diverse degrees of freedom, namely Jahn–Teller activity, orientational ordering of the ammonium molecules, and the formation of hydrogen bonds.

## Introduction

The cooperative Jahn–Teller effect is attributed to structural deformations resulting from the interaction between transition metal ions’ electronic degenerate orbitals and their normal vibration modes.^1^ This interaction reduces the symmetry of the environment surrounding the Jahn–Teller ion, lowering the total energy. Due to their diverse physical properties and structural variations, compounds with Jahn–Teller active ions have gained significant attention in the materials science community. In such compounds, the electron–phonon coupling results in octahedral distortions, favoring the occupation of one of the initially degenerate orbital states.^2^ Concurrently, the choice of orbital state induces orbital ordering, which leads to rich physical behavior.

LaMnO_3_ is an archetypical material with Jahn–Teller active ions.^3^ In this compound, Mn^3+^ has a 3d^4^ electron configuration, which gives rise to Jahn–Teller deformations of the MnO_6_ octahedra. LaMnO_3_ is a parent compound whose derivatives display, e.g., colossal magnetoresistance and polaron confinement.^4,5^ Jahn–Teller active cuprates have also received interest for a long time due to superconductivity.^6,7^

Perovskites, with the chemical formula ABX_3_, can also accommodate other Jahn–Teller-active ions like Cr^2+^ and Cu^2+^ on the B-site, with the electron configurations of 3d^4^ and 3d^9^. Cr^2+^ is isoelectronic to Mn^3+^ but is much less investigated due to its sensitivity to oxidation and elusive chemistry. However, Cr^2+^ can be stabilized in Cr(II) fluoroperovskites, and KCrF_3_ and NaCrF_3_ are discussed in detail in the literature.

KCrF_3_ is a tetragonal Jahn–Teller distorted perovskite with two structural phase transitions: *I*112/*m* to *I*4/*mcm* at 250 K and *I*4/*mcm* to *Pm*3̅*m* at 973 K.^8,9^ The monoclinic and tetragonal phases display Jahn–Teller distortion that stabilizes antiferrodistortive  and  orbital ordering in the *ab*-plane, similar to the *d*-type polymorph of KCuF_3_.^8,10^ The *d*-type polymorph corresponds to a C-type orbital ordering, while the *a*-type polymorph corresponds to a G-type orbital ordering. The cubic phase does not display Jahn–Teller distortions or orbital ordering. Below 79.5 K, KCrF_3_ shows an incommensurate antiferromagnetic ordering, which transitions to a commensurate antiferromagnetic ordering at 45.8 K.^11^ Below 9.5 K, the magnetic structure displays a canting antiferromagnetic ordering and weak ferromagnetism,.^8^

In contrast, NaCrF_3_ (Glazer tilt *a*^–^*b*^–^*c*^–^) is a triclinic fluoroperovskite, crystallizing in space group P1̅.^12,13^ It is highly distorted due to the low tolerance factor induced by the small sodium ion, and no structural phase transitions are reported. Similarly to KCrF_3_ (Glazer tilt *a*^0^*a*^0^*c*^–^) and LaMnO_3_ (Glazer tilt *a*^–^*b*^+^*a*^–^), NaCrF_3_ adopts an A-type antiferromagnetic structure, induced by the orbital ordering scheme driven by the Jahn–Teller effect. However, in contrast to the magnetic structure of KCrF_3_, the magnetic moments in NaCrF_3_ display a slight canting. NaCrF_3_ also displays a metamagnetic transition at 8 T.^12^

This paper presents a new Cr(II) fluoroperovskite, NH_4_CrF_3_, whose the synthesis is briefly described in the literature prepared by an alternative method.^14^ We prepared NH_4_CrF_3_ based on the wet-chemical methods described in ref (12), and here, we present the structural and magnetic properties of NH_4_CrF_3_ investigated by X-ray and neutron diffraction and magnetic measurements.

## Experimental Section

Powder samples of NH_4_CrF_3_ were synthesized according to the procedure established by Bernal et al.,^12,15^ and all work was carried out under strictly inert conditions on a Schlenk line and with degassed solvents. First, chromium(II) acetate dihydrate was prepared from CrCl_3_ × 6H_2_O (Alpha Aesar, 99.5%), which was reduced with zinc (Alpha Aesar, 99.9%) and hydrochloric acid (Fisher Chemical, 37%) to form a Cr(II) solution. Next, chromium(II) acetate was precipitated by adding a supersaturated solution of sodium acetate (Fluka, ≥99.0%). Chromium(II) acetate dihydrate was then washed with H_2_O and acetone (VWR), before it was dried under high vacuum and stored in a glovebox for later use.

NH_4_CrF_3_ was synthesized by adding 8 mL of H_2_O and 5 mL of methanol to 2 g of chromium(II) acetate in a polycarbonate vial and heated to 70 °C. 4 g of NH_4_HF_2_ (Sigma-Aldrich, 99.999%) dissolved in 5 mL of H_2_O was also heated to 70 °C and transferred to the chromium(II) acetate solution under vigorous stirring, and NH_4_CrF_3_ precipitated. The large molar excess (≈13) was used to aid the precipitation of the product. Note that special safety precautions, such as suited PPE and HF-compatible gloves, must be taken when working with bifluorides. The product was washed three times with methanol, filtered, and dried under high vacuum. The final product is stable in air for hours, but for longer storage, a protective atmosphere should be used. Powder X-ray diffraction was collected at room temperature with a Bruker D8 A25 powder diffractometer with Mo radiation and a focusing mirror Dectris Eiger 500R 2D detector, which showed the product to be phase pure. Capillaries were filled in the glovebox and sealed.

High-resolution X-ray diffraction data was collected at the Swiss-Norwegian beamlines BM01 and BM31 at the European Synchrotron Radiation Facility.^16^ Powder samples of NH_4_CrF_3_ were packed in 0.3 mm capillaries in the glovebox, and data was collected with a 2D PILATUS2M detector using a wavelength of 0.73074 Å at BM01 and with a PILATUS3 X CdTe 2 M using a wavelength of 0.25509 Å at BM31. The samples were cooled/heated between 100 and 500 K using an Oxford Cryostream 700+ nitrogen blower. The data was reduced with the Bubble software.^16^ Neutron powder diffraction was collected at the high-resolution powder diffractometer for thermal neutrons (HRPT) at the Swiss Spallation Neutron Source of the Paul Scherrer Institut, Switzerland.^17^ A powder sample of NH_4_CrF_3_ was loaded into a 10 mm vanadium can inside a helium-filled glovebox. Data was collected between 1.7 and 100 K with wavelengths of 1.886 and 1.155 Å. All diffraction data was analyzed with Jana2020^18^ and TOPAS V6.^19^ X-ray data was refined using a peak shape function for powder diffraction on large area detectors.^20^ In the combined refinement, we used neutron data collected at 65 K and X-ray data collected at 100 K and refined the data with individual lattice parameters and common atomic coordinates. The 65 K neutron data was used as the statistics were better, and the data was collected with a shorter wavelength, which is more suited for structural refinements. Bond valence sum calculations were performed according to bond valence = exp((*R*_0_ – *R*)/*B*), with *R*_0_ = 1.67 and *B* = 0.37 for chromium fluoride, *R*_0_ = 1.014 and *B* = 0.413 for hydrogen–nitrogen, and *R*_0_ = 0.708 and *B* = 0.558 for hydrogen fluoride.^21^

Optical measurements were performed on a FLAME-S spectrometer from OceanOptics using a white diode and optical fibers. For the measurements, the samples were filled in 1 mm capillaries and sealed before measurements. The incident light hit the sample surface at a right angle, and the detector was positioned at a 45° angle to it. Absorbance spectra were computed from reflectance using the Kubelka–Munk method.^22^

Magnetic measurements were carried out on a Quantum Design Magnetic Properties Measurement System (MPMS) XL 7T on a powder sample of NH_4_CrF_3_. Temperature-dependent DC magnetic susceptibility χ(*T*) data was measured between 4 and 300 K in a zero-field-cooled/field-cooled (ZFC-FC) mode, under 500 and 5000 Oe fields. Isothermal field-dependent measurements were collected at 4 and 300 K with a maximum field of 50 kOe.

## Results

### Crystal Structure of NH_4_CrF_3_

Powder samples of NH_4_CrF_3_ with a pale blue color were obtained from the wet-chemical synthesis, and evaluation by X-ray powder diffraction data collected at room temperature showed that the samples were phase pure. The diffraction pattern could be indexed in a tetragonal unit cell similar to KCrF_3_.^8^ However, the presence of, e.g., the reflections between 2.2 and 2.8 Å^–1^ indicates that the symmetry deviates from that of both *a*-type and *d*-type polymorphs of KCuF_3_.^10^

**Figure 1:**
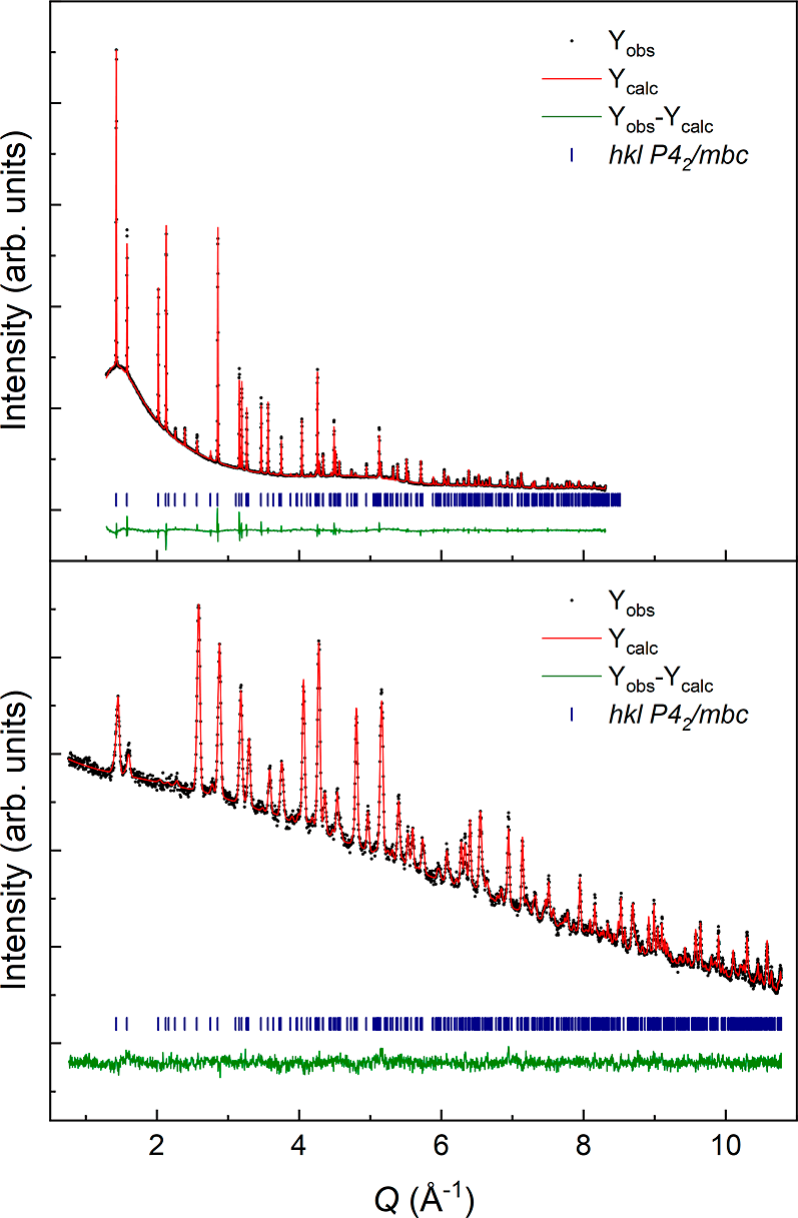
Measured, calculated, and difference curves for combined Rietveld refinement of tetragonal NH_4_CrF_3_ (*P*4_2_/*mbc*) against X-ray (top, λ = 0.25509 Å, 100 K) and neutron powder diffraction (bottom, λ = 1.155 Å, 65 K) data. The first hump in the background for the X-ray data is from the capillary. The sloped background for the neutron powder diffraction data originates from incoherent scattering from hydrogen.

Indexation of the X-ray diffraction data indicates the space group of NH_4_CrF_3_ to be *P*4_2_/*mbc*. Structure solution based on the diffraction data quickly yielded a reasonable solution, however, without hydrogen sites. We found nitrogen to adopt the *4b*-site, which has a −*4* site symmetry, indicating the tetrahedral shape of the NH_4_ groups. Neutron diffraction was employed to determine the hydrogen sites, and we found hydrogen to adopt the *16i*-site around nitrogen. Finally, we performed a combined Rietveld refinement of X-ray (100 K) and neutron (65 K) powder diffraction shown in Figure 1, and the refined structural parameters are given in Table 1. We note that the refined N–H distance of 1.013(3) Å, Table 2, fits well with the expected 1.035(8)–1.045(6) Å in NH_4_F.^23^

**Table 1:** Atomic Coordinates of NH_4_CrF_3_ from Combined Rietveld Refinement of Neutron (λ = 1.155 Å) and X-ray (λ = 0.25509 Å) Data^a^

atom	Wyckoff site	*x*	*y*	*z*	Occ	B_iso_ (Å^2^)
N1	4b	0	0	0.25	1	0.31(3)
H1	16i	0.4176(7)	0.3972(6)	0.3263(4)	1	1.80(5)
Cr1	4c	0	0.5	0	1	0.22(2)
F1	4d	0	0.5	0.25	1	0.43(3)
F2	8h	0.7505(3)	0.2969(2)	0	1	0.51(2)

a The refinement was performed in space group *P*4_2_/*mbc* with individual lattice parameters of *a* = *b* = 6.2257(3) Å and *c* = 7.9654(4) Å for the Neutron data at 65 K and *a* = *b* = 6.22593(6) Å and *c* = 7.96165(8) Å for the X-ray data at 100 K.

**Table 2:** Selected Bond Distances in NH_4_CrF_3_ at 65 K Obtained from Combined Rietveld Refinement of X-ray and Neutron Diffraction Data

bond	distance (Å)	bond	distance (Å)
Cr1–F1	1.9913(5)	H1–F1	2.746(4)
Cr1–F2	2.003(2)	H1–F2	2.885(4)
Cr1–F2	2.418(2)	H1–F2	2.917(4)
N1–H1	1.021(4)	H1–F2	3.049(3)
H1–F2	1.823(4)	H1–F2	3.148(4)
H1–F1	2.598(4)		

**Figure 2:**
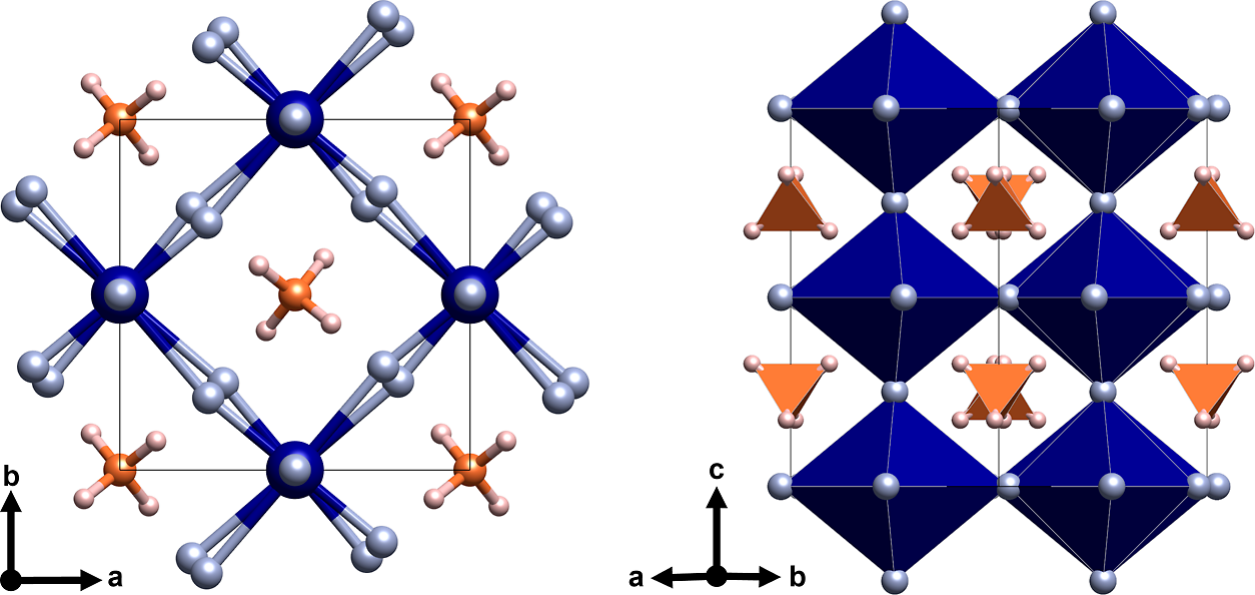
Low-temperature crystal structure of tetragonal NH_4_CrF_3_ (*P*4_2_/*mbc*). Nitrogen, hydrogen, chromium, and fluoride are illustrated as orange, pink, blue, and gray, respectively.

The nuclear structure of NH_4_CrF_3_, Figure 2, is closely related to that of KCrF_3_ and KCuF_3_. The CrF_6_ octahedra have strong Jahn–Teller deformations, with two long and four short Cr–F bonds, corresponding to a pure *Q*_3_ Van Vleck mode, Table 2. The long Cr–F-bonds are in the *ab*-plane and are stacked along the *c*-axis without rotation, corresponding to a Glazer tilt of *a*^0^*a*^0^*c*^–^. The stacking is thus similar to the *d*-type polymorph of KCuF_3_, and NH_4_CuF_3_ is reported to adopt this structure at room temperature.^24^ The stacking of the long Cr–F-bonds corresponds to C-type orbital ordering by analogy to LaMnO_3_.^25^ The difference in symmetry and the crystal structure of NH_4_CrF_3_ and KCrF_3_ has its origin in the anisotropic shape of the NH_4_ cation and hydrogen bonding. Even at room temperature, the NH_4_ groups are ordered and create hydrogen bonds to the fluoride anions in the *ab*-plane (F2 sites). The hydrogen bonds are evident through buckling of the Cr–F–Cr angle, and the Cr1–F2–Cr1 angle is 170.28(7)° at 65 K, Figure 2. The hydrogen bonding is also evident from the bond distances, where one of the H1–F2 bonds is significantly shorter than the other H–F bonds, Table 2. Bond valence sum calculations indicate that chromium is divalent (+1.92), while hydrogen is monovalent (+1.22).

**Figure 3:**
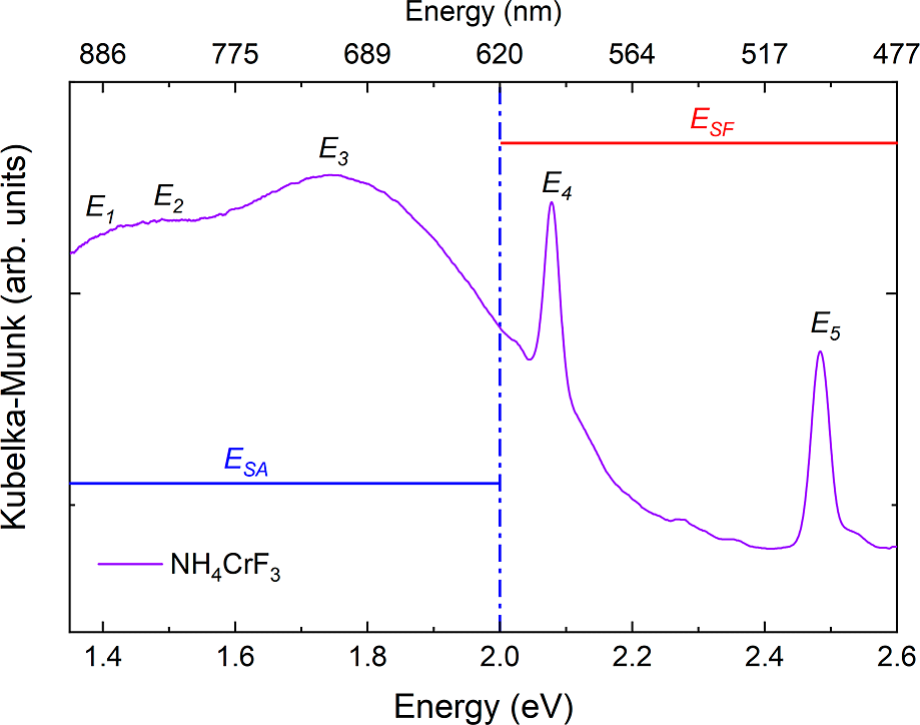
Optical absorption spectra of NH_4_CrF_3_ collected at room temperature. The SA transitions are labeled E_1_–E_3_, while E_4_ and E_5_ are SF.

Optical measurements corroborate the impact of the Jahn–Teller effect and structural distortions on the electronic structure of NH_4_CrF_3_, Figure 3. In the optical absorption spectrum measured at room temperature, we observe three broad levels for the spin-allowed (SA) transitions, *E*_1–3_, between 1.3 and 2 eV. The SA transitions are separated from the two sharp spin-forbidden (SF) transitions *E*_4–5_, which lie between 2 and 2.6 eV. The transitions arise from splitting the *e*_g_ and *t*_2g_ levels by the tetragonal distortion and are thus a signature of the Jahn–Teller distortions. The data is also in good agreement with NaCrF_3_ and KCrF_3_.^13^ This measurement thus confirms (i) the electron configuration of Cr(II), (ii) the presence of the Jahn–Teller distortions, and (iii) the close structural, orbital ordering, and electronic relations to ACrF_3_ with A = Na and K.

### Order–Disorder Transition

**Figure 4:**
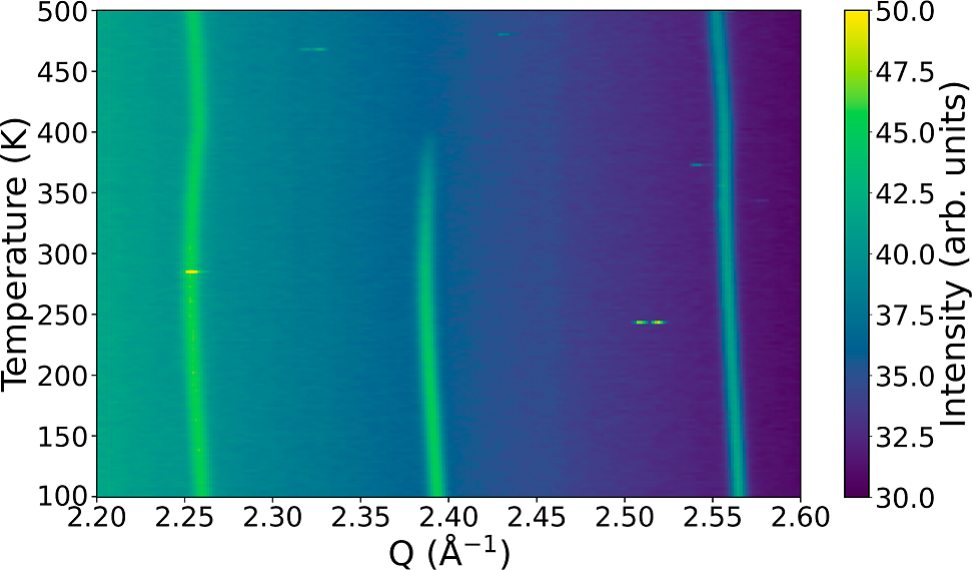
X-ray diffraction patterns of NH_4_CrF_3_ as a function of temperature. The sample was contained in a sealed capillary for the experiment. The (211) reflection at 2.39 Å^–1^ fingerprints the order–disorder transition. The bright dots are cosmic radiation on the detector.

The temperature stability of the ammonium ordering was investigated by X-ray powder diffraction between 100 and 500 K. In the diffraction patterns, the ammonium ordering can be identified by the (2,1,1) reflection at 2.39 Å^–1^. The reflection is a fingerprint of the buckling of the Cr–F–Cr angle, see the Supporting Information. As shown in Figure 4, the reflection disappears around 405 K. Thus, NH_4_CrF_3_ displays an order-to-disorder transition at 405 K. The low-temperature phase has ordered ammonium groups and corresponding buckling of the Cr–F–Cr angle due to hydrogen bonds to fluoride. The disordered high-temperature phase has disordered ammonium groups and no buckling of the Cr–F–Cr angle. The symmetry of the compound changes from *P*4_2_/*mbc* to *P*4/*mbm* in the order-to-disorder transition, corresponding to a transition to higher symmetry. The continuous nature of the transition indicates that it is a second-order transition.

The high-temperature *P*4/*mbm* structure of NH_4_CrF_3_ is identical to that reported for NH_4_CuF_3_ at room temperature and the *d*-type polymorph of KCuF_3_, corresponding to a Glazer tilt of *a*^0^*a*^0^*c*^+^ and C-type orbital ordering.^10^ In the high-temperature phase, the ammonium group can be described with hydrogen in the *16l*-site with 50% occupancy, creating a cube around nitrogen, illustrating the disordered state of the ammonium group at high temperatures. A refinement with anisotropic displacement parameters shows no elongation normal to the bond direction for F2 due to disorder in the buckling of the Cr1–F2–Cr1 angle. Structural information for NH_4_CrF_3_ at 500 K is given in Table 3. Bond valence sum calculations of the high-temperature phase indicate similar oxidation states as the low-temperature phase; chromium is divalent (+1.88), while hydrogen is monovalent (+1.22).

**Table 3:** Atomic Coordinates of NH_4_CrF_3_ from Rietveld Refinement of X-Ray (λ = 0.25509 Å) Data at 500 K in Space Group *P*4/*mbm* with *a* = b = 6.23886(7) Å and c = 4.02014(5) Å^a^

atom	Wyckoff site	*x*	*y*	*z*	Occ	*U*_iso_ (Å^2^)
N1	*2b*	0	0	0.5	1	0.0287(13)
H1	*16l*	0.89376	–0.08089	0.35450	0.5	0.05
Cr1	*2d*	0	0.5	0	1	0.0150(4)
F1	*2c*	0	0.5	0.5	1	0.0239(8)
F2	*4g*	0.2268(4)	0.7268(4)	0	1	0.0284(9)

a Atomic positions, occupation, and thermal displacement parameters for hydrogen were locked to values obtained from neutron diffraction.

To follow the temperature evolution of the ammonium ordering, we performed symmetry-mode analysis through Rietveld refinements generated from ISODISTORT of the ISOTROPY Software Suite.^26,27^ The group–subgroup between *P*4/*mbm* and *P*4_2_/*mbc* is associated with the *Z* point Z  of the Brillouin zone, a basis of {(1, 0, 0), (0, 1, 0), (0, 0, 2)}, and the irreducible representation Z_2_^+^ of *P*4/*mbm*. Two symmetry modes represent the distortions when transitioning from *P*4/*mbm* to *P*4_2_/*mbc*: *a1* representing the displacement of F2 in the *ab*-plane (Z_2_^+^), and *a2* representing the displacement of F along the Cr–F–Cr bond in the *ab*-plane (Γ_1_^+^), i.e., the amplitude of the Jahn–Teller distortion. Hydrogen would follow the Z_2_^+^ occupation mode, which was not included due to the low X-ray contrast. The diffraction data were refined sequentially, and *a1* was restricted to zero above 405 K, i.e., the order-to-disorder transition temperature. The restriction on *a1* was used as the calculated patterns had some calculated intensity at the (211) peak above the transition, although the experimental data showed no intensity.

**Figure 5:**
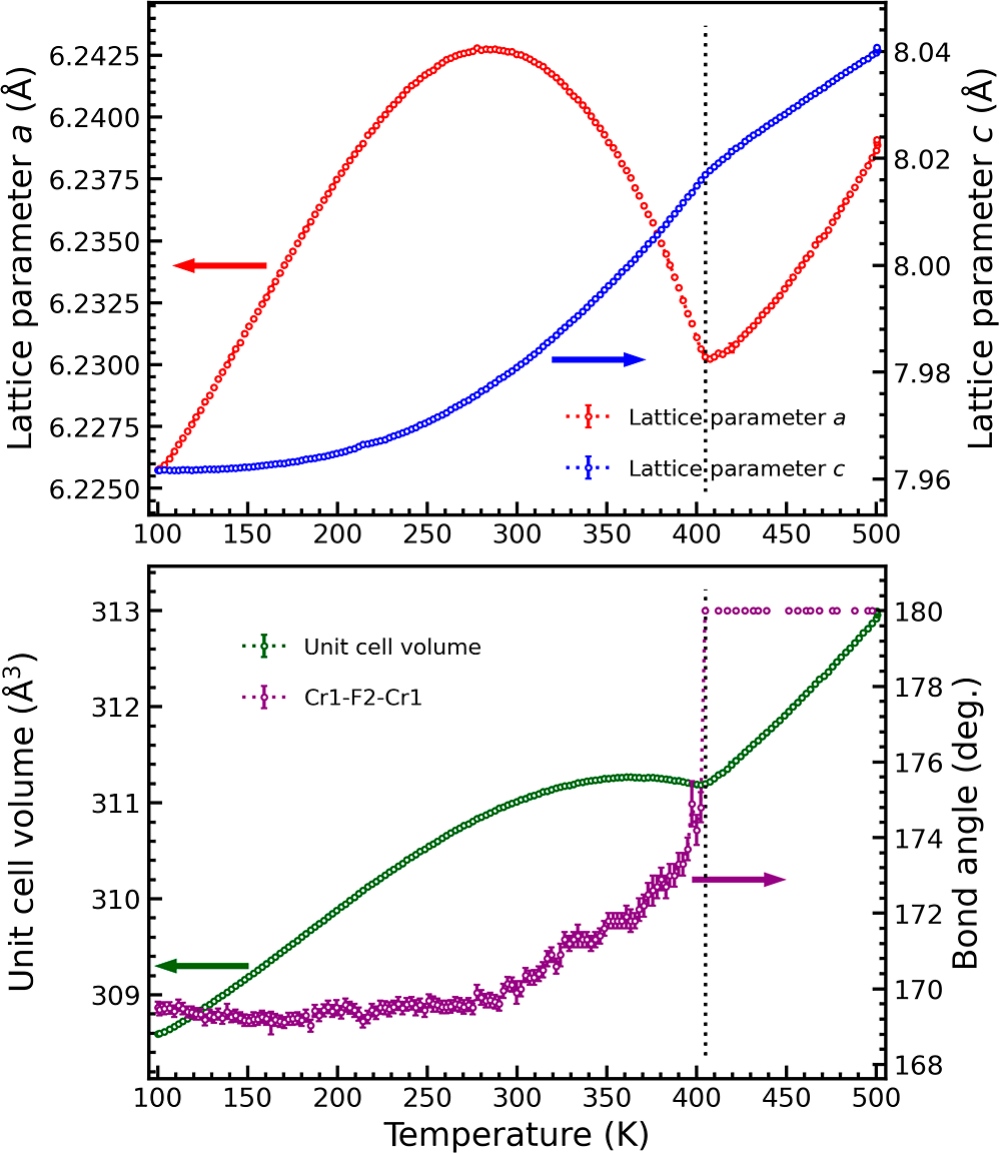
Lattice parameters, unit cell volume, and the Cr1–F2–Cr1 angle from Rietveld refinements for NH_4_CrF_3_ across the ammonium order-to-disorder transition. The dashed line indicates the order-to-disorder transition temperature at 405 K.

Upon heating from 100 K, the lattice parameters and unit cell volume expand linearly as expected, Figure 5. Above 200 K, we observe a change in the slope of the *a* lattice parameter; the expansion decays and the *a* lattice parameters start to contract above 280 K. Simultaneously, the thermal expansion of the *c* lattice parameter escalates. The combination of the contraction of the *a* lattice parameter and the escalating thermal expansion of the *c* lattice parameter reduces the expansion of the unit cell volume, and above 350 K, it contracts slightly. At the order–disorder transition, the thermal expansion changes from negative to positive, and the lattice continues to expand linearly. The changing trends in the thermal expansion can also be directly observed as slight changes in the curvature of the reflections in Figure 4.

In the Rietveld refinements, we refined the *a1* and *a2* symmetry modes, which describe the position of the fluoride atoms. The coordinates of chromium and nitrogen were not refined as they are at special positions, while the coordinates of the hydrogen site were restricted to neutron diffraction values. The changes of the Cr1–F2–Cr1 angle (*a1* symmetry mode) display the most prominent changes, see Figure 5 and the Supporting Information. As *a1* describes the buckling of the Cr1–F2–Cr1 angle, the two parameters follow each other inversely. We observe the Cr1–F2–Cr1 angle to be stable up to 280 K before it starts to increase, coinciding with the change of slope in the thermal expansion of the *a* lattice parameter. The Cr1–F2–Cr1 angle increases and reaches 180° at the order–disorder transition temperature of 405 K. *a2* remains fairly constant throughout the refinements. Thus, the Jahn–Teller ordering remains after the transition. We note that the results from symmetry mode refinements are consistent with Rietveld refinements of the crystal structure without symmetry mode restrictions.

### Magnetic Properties

**Figure 6:**
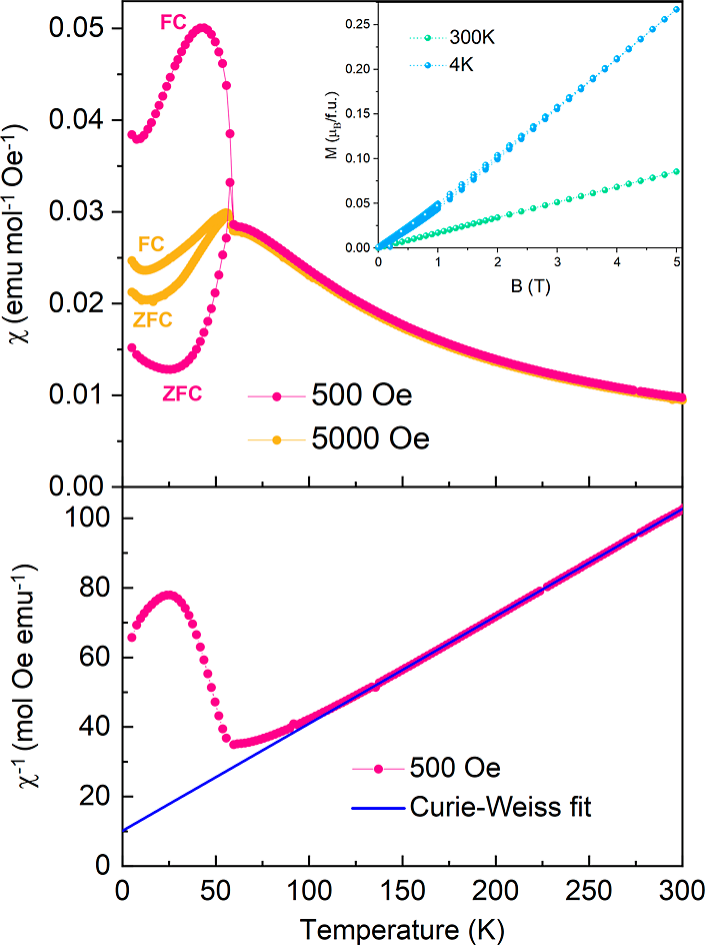
Magnetic measurements of a NH_4_CrF_3_ powder sample. Top: ZFC-FC at 500 and 5000 Oe. Inset: *M*(*H*) curves at 4 and 300 K. Lower: Curie–Weiss fit at 500 Oe of ZFC.

At high temperatures, the DC magnetic susceptibility χ(*T*) of NH_4_CrF_3_ indicates typical paramagnetic behavior, Figure 6. A Curie–Weiss fit at 500 Oe in the temperature range of 100–300 K reveals a paramagnetic moment of μ_eff_ = 4.3 μ_B_, which is in good agreement with the theoretical μ_eff_ = 4.9 μ_B_ for a high-spin Cr^2+^ (*d*^4^) *S* = 2 system, and similar to the μ_eff_ = 4.38 μ_B_ observed in KCrF_3_.^11^ Moreover, the negative Weiss temperature θ = −33 K extracted from the fit in the paramagnetic regime indicates that antiferromagnetic interactions dominate the magnetic moments on Cr^2+^. Isothermal *M*(*H*) curves are also presented in the inset of Figure 6. At 300 K, the magnetization is linear and in agreement with a paramagnetic state.

At low temperatures, the magnetic susceptibility agrees with long-range antiferromagnetic ordering, associated with a Néel temperature of *T*_N_ = 60 K, Figure 6. However, we observe a difference between ZFC and FC below *T*_N_, which is reduced under a larger magnetic field. A similar behavior was observed in KCrF_3_ and attributed to weak ferromagnetism,^8^ while it remains open if this is the case for NH_4_CrF_3_. The *M*(*H*) measurements at 4 K show a tiny opening and low magnetization (0.27 μ_B_/f.u.), which supports this assumption.

### Neutron Diffraction and Magnetic Structure

**Figure 7:**
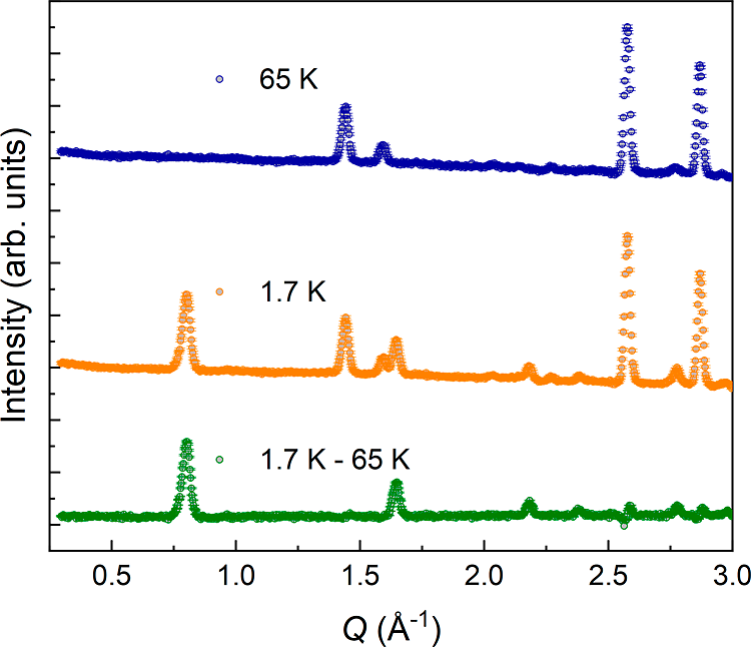
Neutron powder diffraction patterns of NH_4_CrF_3_ measured at HRPT at 1.7 and 65 K, and the difference between the two patterns. The difference curve indicates the reflections originating from magnetic ordering.

Neutron diffraction data collected at HRPT allowed us to investigate the magnetic ordering below the transition temperature in detail. We clearly observe new reflections in the diffraction pattern, most significantly two reflections at 0.78 and 1.63 Å^–1^, which can be indexed as (0,0,1) and (1,1,1) in *P*4_2_/*mbc*, Figure 7. A magnetic propagation vector of *k* = (0, 0, 0) can index the new reflections. The (001) reflection is forbidden in the parent space group, and the magnetic symmetry must thus break the symmetry. The strong intensity of the (001) reflection also indicates that the magnetic moments have a significant component in the *ab*-plane. Considering these indices in the aristotype perovskite setting (*Pm*3̅*m* basis  with respect to *P*4_2_/*mbc*), we can index these peaks as  and , indicating A-type magnetic ordering, which is the *X*-point irreducible representation with respect to *Pm*3̅*m*.

Based on the *k* = (0, 0, 0) propagation vector, we evaluate the possible magnetic space groups and irreducible representations associated with the parent space group *P*4_2_/*mbc* at the gamma point Γ [0,0,0] of the Brillouin zone. We find the magnetic space groups *Ccc*′*m*′, *Pb*′*am*′, and *P*2′/*m*′, which belong to the m Γ_5_^+^ irreducible representation of *P*4_2_/*mbc*, to describe the data accurately. The three magnetic space groups yield a similar fit and magnetic ordering. We can thus not distinguish the magnetic space groups from each other based on the data. We note that *Ccc*′*m*′ and *P*2′/*m*′ both allow a ferromagnetic component, while *Pb*′*am*′ does not. The three magnetic space groups all have *M*_*z*_ = 0 by symmetry.

*Ccc*′*m*′ and *Pb*′*am*′ have two independent chromium sites, while *P*2′/*m*′ has four. Our refinements show that the sites converge toward the same ordered moment with opposite signs. No improvement is observed when the moments are refined individually compared to when they are restricted to the same value. We thus continue with restricting the moments to the same amplitude.

The magnetic space groups *Ccc*′*m*′, *Pb*′*am*′, and *P*2′/*m*′ allow the moments to order in different directions. From powder neutron diffraction, we cannot determine the direction of the magnetic moments except for their angle with the unique axis of the magnetic structure as the symmetry is uniaxial.^28^ We thus continue our analysis with *Ccc*′*m*′ (BNS: 66.496) and its symmetry restrictions, as this magnetic space group has the highest symmetry.

The magnetic unit cell of *Ccc*′*m*′ has a basis {(−1, −1, 0), (−1, 1, 0), (0, 0, 1)} with respect to *P*4_2_/*mbc* and a shift of . In the refinements, we find that *M*_*y*_ dominates the magnetic signal. *M*_*x*_ represents a ferromagnetic canting of the moments and was restricted to zero as it does not significantly influence the calculated diffraction pattern. It is thus evident that if a ferromagnetic component is present in the neutron diffraction, it is too small to be observed with the available data. The final Rietveld refinement of the magnetic structure and structural information are shown in the Supporting Information.

**Figure 8:**
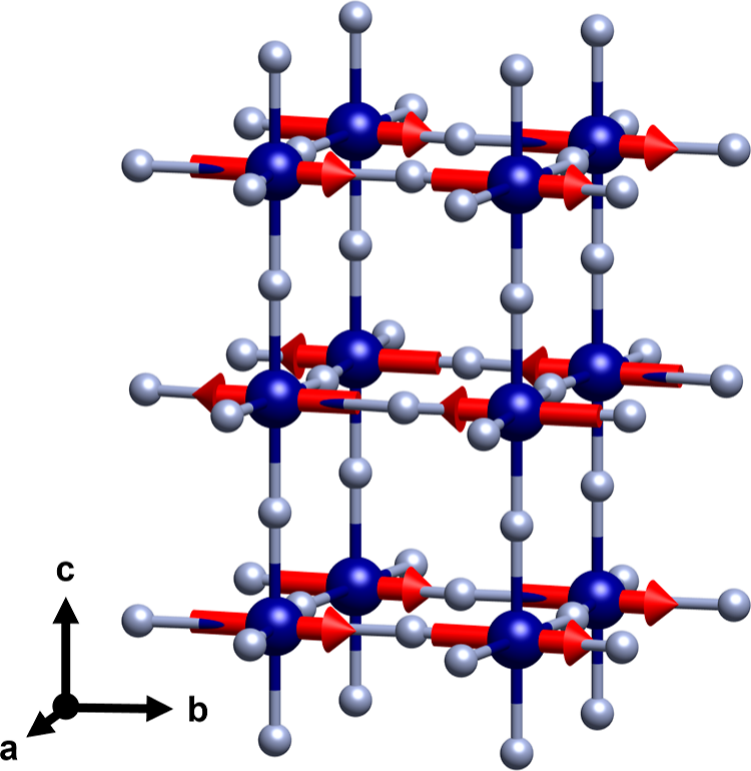
Magnetic structure of NH_4_CrF_3_ with magnetic space group *Ccc*′*m*′ with a basis {(−1, −1, 0), (−1, 1, 0), (0, 0, 1)} and a shift of  from the original cell. The magnetic moments of the chromium atoms form ferromagnetic layers that are stacked antiferromagnetic, yielding A-type antiferromagnetic structure. Chromium atoms are shown in blue and fluorine in gray. The ammonium groups are removed for clarity.

The magnetic structure is illustrated in Figure 8 and can be described as an A-type antiferromagnetic structure. The magnetic moments construct ferromagnetic layers with the moments directed along the -direction in the original cell, corresponding to [0,1,0] in the new basis. The ferromagnetic layers are stacked in an antiferromagnetic manner along [0,0,1]. The magnetic moments are thus directed along the Cr1–F2 bonds. The refined magnetic moment is 3.72(2) μ_B_ at 1.7 K, which is in excellent agreement with the expected value of 4 μ_B_. Temperature dependence of the magnetic moment and the lattice parameters can be found in the Supporting Information.

## Discussion

Here, we present a new member of the elusive ACrF_3_ fluoroperovskites based on a robust and reliable wet-chemical synthesis method. Again, this proves that the wet-chemical approach allows the preparation of Cr(II) fluoroperovskites, despite their sensitivity to oxidation, and ACrF_3_ with A = Na, K, and NH_4_ are now reported with this method.^12,13^ Interestingly, we observe three different crystal structures at room temperature for A = Na^+^, K^+^, and NH_4_^+^. The deviations in crystal structure induced by different A-sites also cause slight differences in the physical properties of the compounds. However, the similarity in the electronic structure is evident from optical measurements.

The crystal structure of NH_4_CrF_3_ has the same arrangement of the long Jahn–Teller axis as the *d*-type polymorph of KCuF_3_, corresponding to the same antiferrodistortive orbital ordering of  and  in the *ab*-plane and C-type orbital ordering. The structure also stands out among the ammonium fluoroperovskites as it displays ammonium ordering at room temperature, in contrast to, e.g., NH_4_MnF_3_, NH_4_CoF_3_, and NH_4_CuF_3_.^29−32^ The ordering temperature of 405 K is significantly higher than that of NH_4_MnF_3_ and NH_4_CoF_3_, of 182 and 127.7 K, respectively,^29,31^ indicating that the hydrogen bonding is particularly strong in NH_4_CrF_3_.

In addition to the ammonium ordering, NH_4_CrF_3_ displays buckling of the Cr–F–Cr angle, indicative of H–F bonding. Such buckling of the bonds is not reported for the low-temperature phase of NH_4_CoF_3_, while it is observed in NH_4_MnF_3_. However, while the buckling in NH_4_MnF_3_ has a displacement in all three directions, the buckling in NH_4_CrF_3_ corresponds to the displacement of fluorine in the *ab*-plane. The buckling of the Cr–F–Cr angle is however similar in NH_4_CrF_3_ and NH_4_MnF_3_, with 170° and 169–174°, respectively.^29^

Hydrogen bonding in halide perovskites is often observed in hybrid halide perovskites. The strength of the hydrogen bond is indicated by the order-to-disorder transition temperature.^33^ Although we have not experimentally estimated the hydrogen-bonding energies, the high order-to-disorder transition temperature indicates that the strength of the hydrogen bonds in NH_4_CrF_3_ is strong, also compared to, e.g., hybrid halide perovskites. For example, AmBX_3_ with Am^+^ = hydrazinium (NH_2_NH^3+^) and guanidinium  have ordering temperatures order-to-disorder transition temperatures of 352 and 503 K and hydrogen-bonding energies of 0.28 and 0.21 eV, respectively.^33^

The complexity of the structural chemistry of NH_4_CrF_3_ results from diverse degrees of freedom, namely, Jahn–Teller activity, orientational ordering of the ammonium molecules, and the formation of hydrogen bonds. It is clear that the energy scale of the Jahn–Teller ordering is the largest. Ammonium groups are typically disordered above 100 K in fluoroperovskites, and we thus estimate the energy scale of the hydrogen bonding to be larger than that of the rotation of ammonium molecules. Based on the current data, we cannot estimate the energy scale of these processes further.

We believe that the high ordering temperature of NH_4_CrF_3_ can be attributed to the Jahn–Teller bonds’ susceptibility to distortions. The increased flexibility is, e.g., demonstrated in hybrid organic–inorganic perovskites.^34^ Another example is KCuF_3_ under high pressure; the Jahn–Teller axis is compressed more than the other axes.^35^ The increased flexibility of the Jahn–Teller and hydrogen bonding leads to the buckling of the Cr–F–Cr angle, and the flexibility allows the hydrogen bonds to stabilize the ammonium-ordered phase at higher temperatures. These results showcase how different *A*-site cations can influence the crystal structure of fluoroperovskites.

Unlike NH_4_CrF_3_, NH_4_CuF_3_ does not display ammonium ordering at room temperature. Although the ordering transition temperature of NH_4_CuF_3_ is unknown, it is certainly below room temperature. This indicates that there is a difference between the two compounds. The quadratic elongation, λ (, where *l*_0_ is the center-to-vertex distance), is larger for NH_4_CuF_3_, thus not the origin of the different ordering temperatures. We suggest that the origin is the smaller size of copper(II) compared to chromium(II), which yields a larger tolerance factor that makes NH_4_CuF_3_ less susceptible to deformations.

The contraction of the unit cell is attributed to the order–disorder transition of the ammonium groups. When heated from 100 K, the sample undergoes normal thermal expansion. Above 200 K, the slope of the *a*-axis starts gradually to change, Figure 5, and we believe that hydrogen bonds start to be broken in this temperature region. This leads to a contraction of the *a*-axis above 280 K due to the contraction of the Cr–F2 bonds that counteract the straining of the Cr1–F2–Cr1 angle. The effect is parallel to the ice-to-water transition, which is also associated with a volume reduction. A volume contraction is also observed in the tetragonal to the cubic phase transition of KCrF_3_.^9^ However, this is an orbital melting transition and not an order-to-disorder transition. We also note that the unit cell volume of NH_4_CrF_3_ is larger than expected based on the size of the ammonium cation compared to KCrF_3_. This is explained by hydrogen bonds deforming the structure and increasing the volume.

For the indications of a second-order transition, it is easy to envision the continuous evolution of the octahedral tiling. However, it is more natural to envision a first-order transition for the ammonium freezing. We note that X-ray data was used to monitor the transition, and we extracted information about the unit cell dimensions. Although the octahedral distortions appear as second order, we do not probe the ammonium groups, and a technique like quasi-elastic neutron scattering would be more suited to address such aspects of the transition.

The A-type antiferromagnetic structure of NH_4_CrF_3_ obtained from neutron diffraction is in good agreement with theoretical expectations from the Goodenough–Kanamori–Anderson rules^36^ based on the C-type orbital ordering scheme: ferromagnetic exchange between  and  and antiferromagnetic exchange between  and  orbitals. Neutron diffraction studies of NaCrF_3_ and KCrF_3_ also suggest that an A-type antiferromagnetic structure should be expected.^11,12^ A-type antiferromagnetism is also in agreement with the isoelectronic Mn^3+^ manganite perovskite and the related KCuF_3_.^10,37^

In contrast to the case of KCrF_3_, we did not observe any incommensurate component of the magnetic propagation vector and no tilting of the magnetic moments as observed in NaCrF_3_.^12^ However, as in KCrF_3_, we observe similar indications of weak ferromagnetism in the susceptibility data, which display a difference between ZFC and FC that can be reduced with increasing magnetic field.^8^ The neutron diffraction analysis shows that a ferromagnetic canting of the spins away from a collinear A-type antiferromagnetic configuration is allowed by symmetry for *Ccc*′*m*′ and *P*2′/*m*′ but not for *Pb*′*am*′. The neutron diffraction does not give experimental support for canting. By a very crude approximation, the tilting angle can be estimated from the *M*(*H*) measurements at 4 K to  at 4 T external field. As this angle should be smaller at lower fields, it is unlikely to be observable with powder neutron diffraction, especially with the incoherent background from hydrogen. It thus remains open whether the indications of weak ferromagnetism are intrinsic to the system.

A small amount of ferromagnetic impurity may also explain the difference between ZFC and FC. The small increase in susceptibility at very low temperatures would also support this hypothesis. However, no secondary phase was identified during our structural analysis, evidencing the high purity of the sample.

## Conclusions

For the first time, the structural chemistry of NH_4_CrF_3_ is investigated in detail. It adopts a tetragonal Jahn–Teller distorted perovskite crystal structure with *d*-type stacking of the Jahn–Teller bonds and C-type orbital ordering. At room temperature, the ammonium groups order and display clear signs of hydrogen bonds to nearby fluoride atoms by buckling the Cr–F–Cr angle away from 180°. Our results indicate a correlation between the flexibility of the Jahn–Teller ion, the hydrogen bond formation, and the ammonium cation ordering. The Jahn–Teller effect makes the bonds more susceptible to distortions, allowing hydrogen bonds to stabilize an ammonium-ordered structure up to 405 K. Across the order-to-disorder transition, we observe a unit cell contraction associated with breaking hydrogen bonds, similar to the phenomenon observed in water ice melting. NH_4_CrF_3_ is an A-type antiferromagnet with an Neél temperature of 60 K, in agreement with the C-type orbital ordering scheme and other Cr(II) fluoroperovskites.

## Supporting Information Available

Simulated and experimental diffraction patterns of the ordered and disordered phases; refined parameters, bonds, and angles from sequential Rietveld refinements; illustration of symmetry modes; examples of Rietveld refinements from sequential Rietveld refinements; structural information and Rietveld refinement for the magnetic structure; lattice parameters and magnetic moment as a function of temperature; and magnetic (001) reflection as a function of temperature from neutron diffraction (PDF)

## References

[ref1] Jahn H. A.; Teller E. Stability of polyatomic molecules in degenerate electronic states - I - Orbital degeneracy. Proc. R. Soc. A 1937, 161, 220–235.

[ref2] Sturge M. D. The Jahn-Teller Effect in Solids. Solid State Phys. 1968, 20, 91–211. 10.1016/S0081-1947(08)60218-0.

[ref3] Mahendiran R.; Tiwary S. K.; Raychaudhuri A. K.; Ramakrishnan T. V.; Mahesh R.; Rangavittal N.; Rao C. N. R. electron-transport properties, and giant magnetoresistance of hole-doped LaMnO_3_ systems. Phys. Rev. B 1996, 53, 3348–3358. 10.1103/physrevb.53.3348. 9983844

[ref4] Rønnow H. M.; Renner Ch.; Aeppli G.; Kimura T.; Tokura Y. Polarons and confinement of electronic motion to two dimensions in a layered Manganite. Nature 2006, 440, 1025–1028. 10.1038/nature04650. 16625191

[ref5] De Teresa J. M.; Ibarra M. R.; Algarabel P.; Morellon L.; García-Landa B.; Marquina C.; Ritter C.; Maignan A.; Martin C.; Raveau B.; Kurbakov A.; Trounov V. Magnetic versus orbital polarons in colossal magnetoresistance manganites. Phys. Rev. B 2002, 65, 100403(R) 10.1103/physrevb.65.100403.

[ref6] Keller H.; Bussmann-Holder A.; Müller K. A. Ml̆ler Jahn–Teller physics and high-*T*_c_ superconductivity. Mater. Today 2008, 11, 38–46. 10.1016/s1369-7021(08)70178-0.

[ref7] Murphy D. W.; Sunshine S.; van Dover R. B.; Cava R. J.; Batlogg B.; Zahurak S. M.; Schneemeyer L. F. New superconducting cuprate perovskites. Phys. Rev. Lett. 1987, 58, 1888–1890. 10.1103/PhysRevLett.58.1888. 10034564

[ref8] Margadonna S.; Karotsis G. Cooperative Jahn-Teller Distortion, Phase Transitions, and Weak Ferromagnetism in the KCrF_3_ Perovskite. J. Am. Chem. Soc. 2006, 128, 16436–16437. 10.1021/ja0669272. 17177357

[ref9] Margadonna S.; Karotsis G. High temperature orbital order melting in KCrF_3_ perovskite. J. Mater. Chem. 2007, 17, 2013–2020. 10.1039/b700487g.

[ref10] Kurogi H. n. Y.; Kurogi Y. One-Dimensional Antiferromagnetic Properties of KCuF_3_. Prog. Theor. Phys. Suppl. 1970, 46, 147–161. 10.1143/ptps.46.147.

[ref11] Xiao Y.; Su Y.; Li H.-F.; Kumar C. M. N.; Mittal R.; Persson J.; Senyshyn A.; Gross K.; Brueckel T. Th. Brueckel Neutron diffraction investigation of the crystal and magnetic structures in KCrF_3_ perovskite. Phys. Rev. B 2010, 82, 094437 10.1103/physrevb.82.094437.

[ref12] Bernal F. L. M.; Sottmann J.; Wragg D. S.; Fjellvåg H.; Fjellvåg Ø. S.; Drathen C.; Sławiński W. A.; Løvvik O. M. Structural and magnetic characterization of the elusive Jahn-Teller active NaCrF_3_. Phys. Rev. Mater. 2020, 4, 054412 10.1103/PhysRevMaterials.4.054412.

[ref13] Bernal F. L. M.; Lundvall F.; Kumar S.; Hansen P.-A. S.; Wragg D. S.; Fjellvåg H.; Løvvik O. M. Jahn-Teller active fluoroperovskites *A*CrF_3_ (*A* = Na^+^, K^+^): Magnetic and thermo-optical properties. Phys. Rev. Mater. 2021, 5, 064420 10.1103/PhysRevMaterials.5.064420.

[ref14] Earnshaw A.; Larkworthy L. F.; Patel K. S. Chromium(II) chemistry. Part II. Chromofluorides. J. Chem. Soc. A 1966, 363–365. 10.1039/j19660000363.

[ref15] Bernal F. L. M.; Gonano B.; Lundvall F.; Wragg D. S.; Fjellvåg H.; Veillon F.; Sławiński W. A.; Fjellvåg Ø. S. g Canted antiferromagnetism in high-purity NaFeF_3_ prepared by a novel wet-chemical synthesis method. Phys. Rev. Mater. 2020, 4, 114412 10.1103/physrevmaterials.4.114412.

[ref16] Dyadkin V.; Pattison P.; Dmitriev V.; Chernyshov D. A new multipurpose diffractometer PILATUS*@*SNBL. Synchrotron Radiat. 2016, 23, 825–829. 10.1107/S1600577516002411. 27140164

[ref17] Fischer P.; Frey G.; Koch M.; Könnecke M.; Pomjakushin V.; Schefer J.; Thut R.; Schlumpf N.; Bürge R.; Greuter U.; Bondt S.; Berruyer E. High-resolution powder diffractometer HRPT for thermal neutrons at SINQ. Phys. B 2000, 276–278, 146–147. 10.1016/S0921-4526(99)01399-X.

[ref18] Petříček V.; Dušek M.; Palatinus L. Crystallographic Computing System JANA2006: General features. Z. Kristallogr.—Cryst. Mater. 2014, 229, 345–352. 10.1515/zkri-2014-1737.

[ref19] Coelho A. A. *TOPAS* and *TOPAS-Academic*: an optimization program integrating computer algebra and crystallographic objects written in C++. J. Appl. Crystallogr. 2018, 51, 210–218. 10.1107/s1600576718000183.

[ref20] Chernyshov D.; Dyadkin V.; Emerich H.; Valkovskiy G.; McMonagle C. J.; van Beek W. On the resolution function for powder diffraction with area detectors. Acta Crystallogr. 2021, 77, 497–505. 10.1107/S2053273321007506. 34473102

[ref21] Brown I. D. The Chemical Bond in Inorganic Chemistry: The Bond Valence Model; Oxford University Press, 2002; .

[ref22] Alcaraz de la Osa R.; Iparragirre I.; Ortiz D.; Saiz J. M. The extended Kubelka–Munk theory and its application to spectroscopy. ChemTexts 2020, 6, 2 10.1007/s40828-019-0097-0.

[ref23] Adrian H. W. W.; Feil D. The structure of NH_4_F as determined by neutron and X-ray diffraction. Acta Crystallogr., Sect. A: Cryst. Phys., Diffr., Theor. Gen. Crystallogr. 1969, 25, 438–444. 10.1107/S056773946900088X.

[ref24] Troyanov S. I.; Morozov I. V.; Korenev Y. M. The synthesis and crystal structure of ammonium fluorocuprates NH_4_CuF_3_ and (NH_4_)_2_CuF_4_. Russ. J. Inorg. Chem. 1993, 38, 909–913.

[ref25] Hotta T.; Yunoki S.; Mayr M.; Dagotto E. A-type antiferromagnetic and C-type orbital-ordered states in LaMnO_3_ using cooperative Jahn-Teller phonons. Phys. Rev. B 1999, 60, R15009(R) 10.1103/physrevb.60.r15009.

[ref26] Stokes H. T.; Hatch D. M.; Campbell B. J. ISODISTORT, ISOTROPY Software Suite; Brigham Young University.

[ref27] Campbell B. J.; Stokes H. T.; Tanner D. E.; Hatch D. M. ISODISPLACE: a web-based tool for exploring structural distortions. J. Appl. Crystallogr. 2006, 39, 607–614. 10.1107/S0021889806014075.

[ref28] Shirane G. A note on the magnetic intensities of powder neutron diffraction. Acta Crystallogr. 1959, 12, 282–285. 10.1107/S0365110X59000871.

[ref29] Laguna M. A.; Sanjuan M. L.; Orera V. M.; Rubin J.; Palacios E.; Pique M. C.; Bartolome J.; Berar J. F. X-ray and Raman study of the low temperature NH_4_MnF_3_ structure; evidence of librational motion of the NH_4_^+^ ion. J. Phys.: Condens. Matter 1993, 5, 283–300. 10.1088/0953-8984/5/3/005.

[ref30] Rubin J.; Palacios E.; Bartolome J.; Rodriguez-Carvajal J. A single-crystal neutron diffraction study of NH_4_MnF_3_. J. Phys.: Condens. Matter 1995, 7, 563–575. 10.1088/0953-8984/7/3/011.

[ref31] Siebeneichler S.; Dorn K. V.; Smetana V.; Valldor M.; Mudring A. V. A soft chemistry approach to the synthesis of single crystalline and highly pure (NH_4_)CoF_3_ for optical and magnetic investigations. J. Chem. Phys. 2020, 153, 104501 10.1063/5.0023343. 32933281

[ref32] Crocket D. S.; Haendler H. M. Synthesis of Fluorometallates in Methanol. Some Structure Relationships^1a^. J. Am. Chem. Soc. 1960, 82, 4158–4162. 10.1021/ja01501a009.

[ref33] Svane K. L.; Forse A. C.; Grey C. P.; Kieslich G.; Cheetham A. K.; Walsh A.; Butler K. T. How Strong Is the Hydrogen Bond in Hybrid Perovskites?. J. Phys. Chem. Lett. 2017, 8, 6154–6159. 10.1021/acs.jpclett.7b03106. 29216715 PMC5765532

[ref34] Gui D.; Ji L.; Muhammad A.; Li W.; Cai W.; Li Y.; Li X.; Wu X.; Lu P. Jahn–Teller Effect on Framework Flexibility of Hybrid Organic–Inorganic Perovskites. J. Phys. Chem. Lett. 2018, 9, 751–755. 10.1021/acs.jpclett.7b03229. 29360368

[ref35] Zhou J.-S.; Alonso J. A.; Han J. T.; Fernández-Díaz M.; Cheng J.-G.; Goodenough J. B. Jahn–Teller distortion in perovskite KCuF_3_ under high pressure. J. Fluorine Chem. 2011, 132, 1117–1121. 10.1016/j.jfluchem.2011.06.047.

[ref36] Goodenough J. B. Magnetism and the Chemical Bond; R. E. Krieger Publishing Company, 1976; .

[ref37] Solovyev I.; Hamada N.; Terakura K. Zone Boundary Softening of the Spin-Wave Dispersion in Doped Ferromagnetic Manganites. Phys. Rev. Lett. 1996, 76, 4825–4828. 10.1103/PhysRevLett.76.4825. 10061390

