# Supplementary material for: Order-to-Disorder Transition and Hydrogen Bonding in the Jahn–Teller Active NH4CrF3 Fluoroperovskite
Source: Inorg Chem. 2024 May 24;63(23):10594–602. doi: 10.1021/acs.inorgchem.4c00931 (PMC11167635; doi:10.1021/acs.inorgchem.4c00931)
Supplement: Supplementary file 1 — ic4c00931_si_001.pdf [file ic4c00931_si_001.pdf]

Order-to-disorder transition and hydrogen bonding in the Jahn-Teller active NH_4CrF_3 fluoroperovskite

Øystein S. Fjellvåg,[†,‡] Bruno Gonano,[¶] Fabian L. M. Bernal,[§] Salah B. Amedi,[¶] Jike Lyu,[‖] Vladimir Pomjakushin,[‡] Marisa Medarde,[‖] Dmitry Chernyshov,[⊥] Kenneth Marshall,[⊥] Martin Valldor,[¶] Helmer Fjellvåg,[*,¶] and Bjørn C. Hauback[†]

†*Department for Hydrogen Technology, Institute for Energy Technology, PO Box 40, NO-2027, Kjeller, Norway*

‡*Laboratory for Neutron Scattering and Imaging, Paul Scherrer Institute, CH-5232 Villigen-PSI, Switzerland*

¶*Chemistry Department and Center for Material Science and Nanotechnology, University of Oslo, NO-0315, Norway*

§*Division for Research, Dissemination and Education, IT-department, University of Oslo, Oslo, Norway*

‖*Laboratory for Multiscale Materials Experiments, Paul Scherrer Institut, CH-5232 Villigen-PSI, Switzerland*

⊥*Swiss-Norwegian Beam Lines at European Synchrotron Radiation Facility, 71 Avenue des Martyrs, 38043 Grenoble, France*

E-mail: helmer.fjellvag@kjemi.uio.no

Supplementary Material

Buckling of the Cr-F-Cr angle

Figure S1 illustrates the measured and simulated diffraction patterns of the ammonium-ordered and disordered structures. It is evident that the reflection at 2.39 Å^{-1} is a fingerprint of the ammonium-ordered phase. Beyond this difference, there are no clear differences in the diffraction patterns of the ammonium-ordered and disordered structures.

Figure S2 and Figure S3 show the distortion mode amplitudes and selected bond lengths and angles obtained in the Rietveld refinements, while Figure S4 illustrates the distortions in the crystal structure. Examples of of Rietveld refinements from the sequential refinements are shown for 100 K in Figure S5, and 500 K in Figure S6.

In the sequential refinements, the refined patterns showed that the refinements did not properly capture the transition from $P4_2/mbc$ to $P4/mbm$. Instead, a slight amplitude on a1 was maintained, yielding a slight distortion of the Cr1-F2-Cr1 angle and intensity of the (211)-peak, which was inconsistent with the diffraction patterns. To counteract this, the amplitude of a1 was restricted to zero above 405 K, coinciding with the ordering temperature.

Neutron diffraction

To complement the results presented in the main text, we here show the refined data at 1.7 K in Figure S7. Information about the magnetic structure is given in Table 1. The evolution through temperature of the lattice parameters is shown in Figure S8, the magnetic moment in Figure S9, and the (001) reflection in Figure S10.

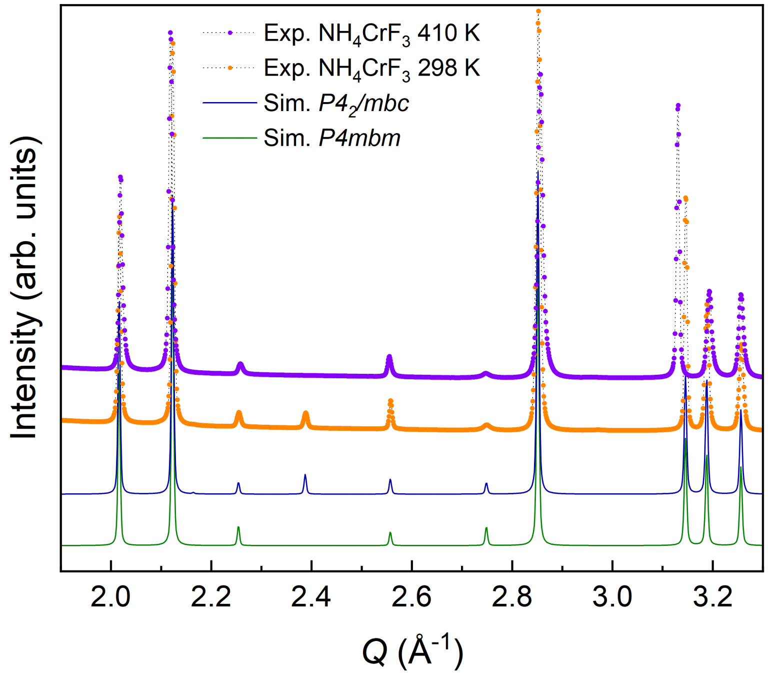

Figure S1: Experimental and simulated diffraction patterns above and below the order-to-disorder transition. The reflection at 2.39 Å^{-1} indicates the ordering of the ammonium groups.

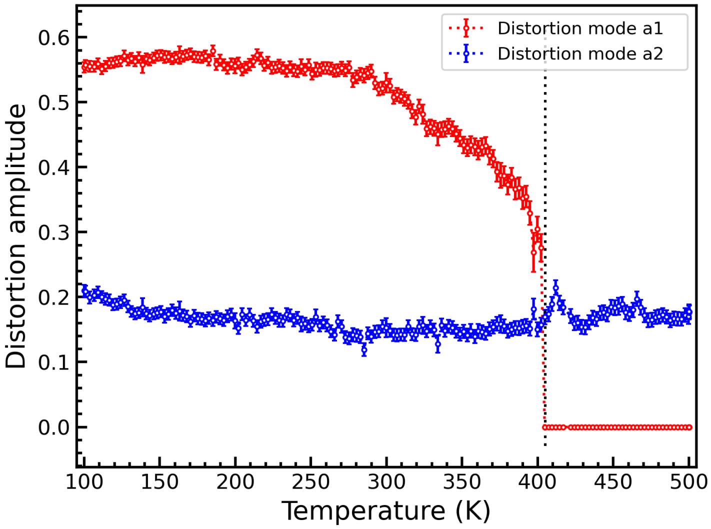

Figure S2: The amplitude of the symmetry mode *a1* (red) and *a2* (blue) extracted from Rietveld refinements.

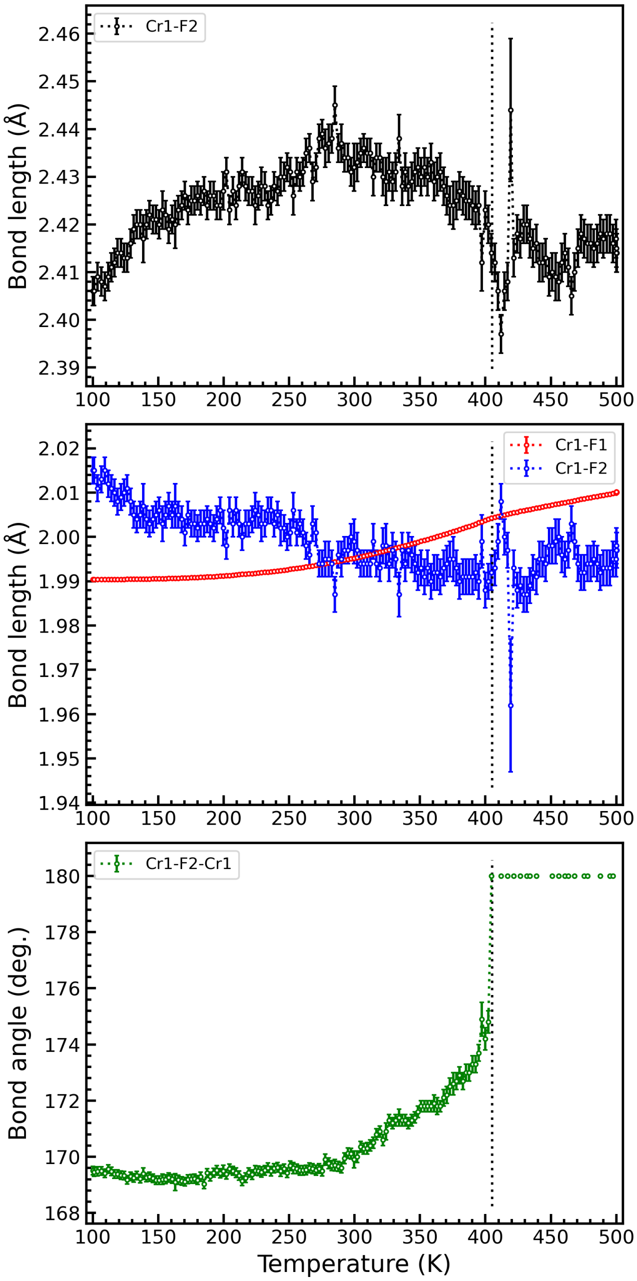

Figure S3: Selected bond lengths and angles for NH_4CrF_3 extracted from Rietveld refinements.

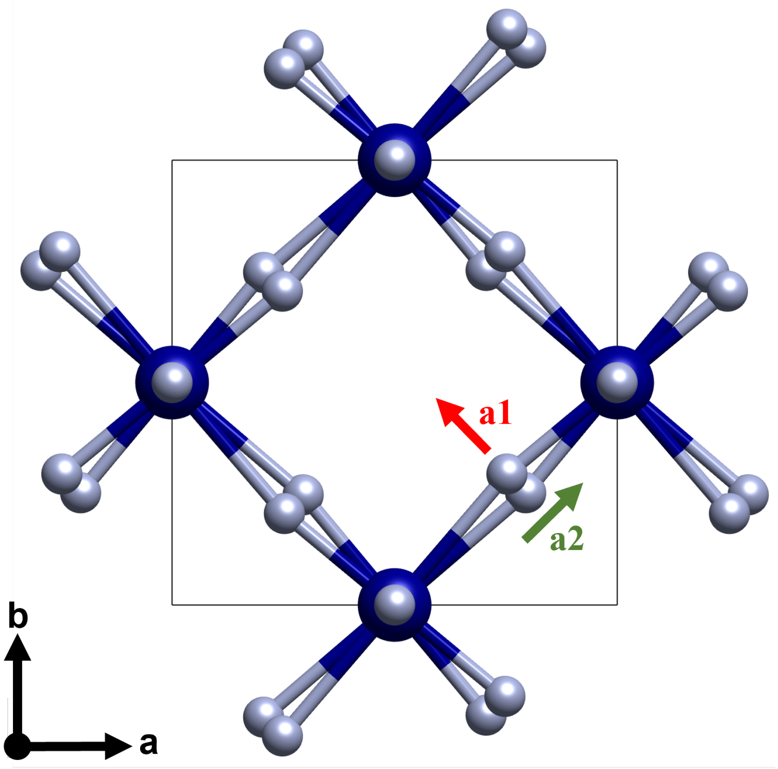

Figure S4: Illustration of the distortion modes in the low temperature structure of NH_4CrF_3. The red and green arrows correspond to the *a1* and *a2* symmetry modes, respectively. The ammonium groups are omitted for clarity.

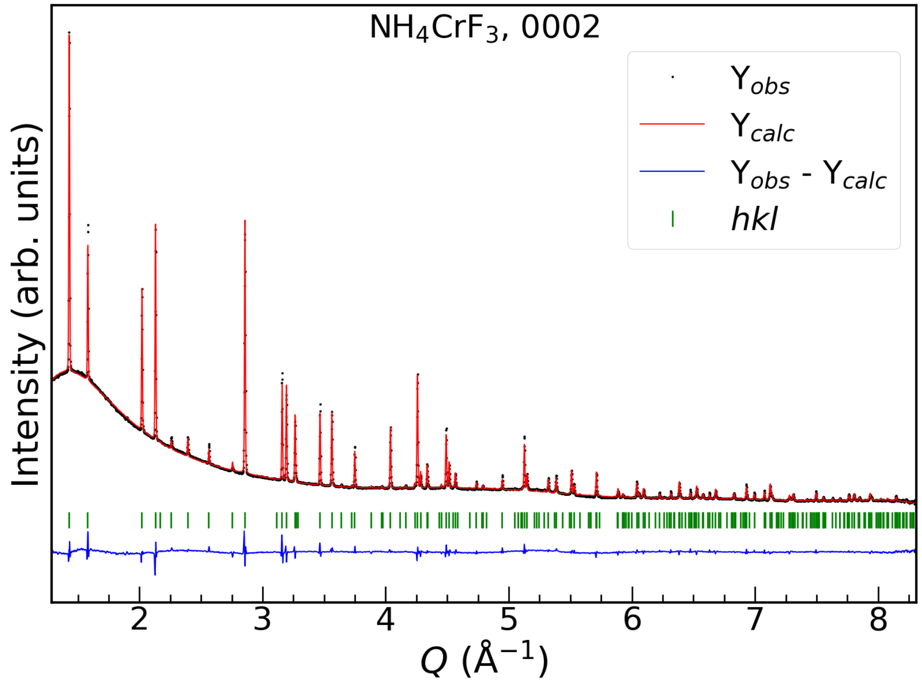

Figure S5: Rietveld refinement of NH_4CrF_3 data collected at BM31 at 100 K.

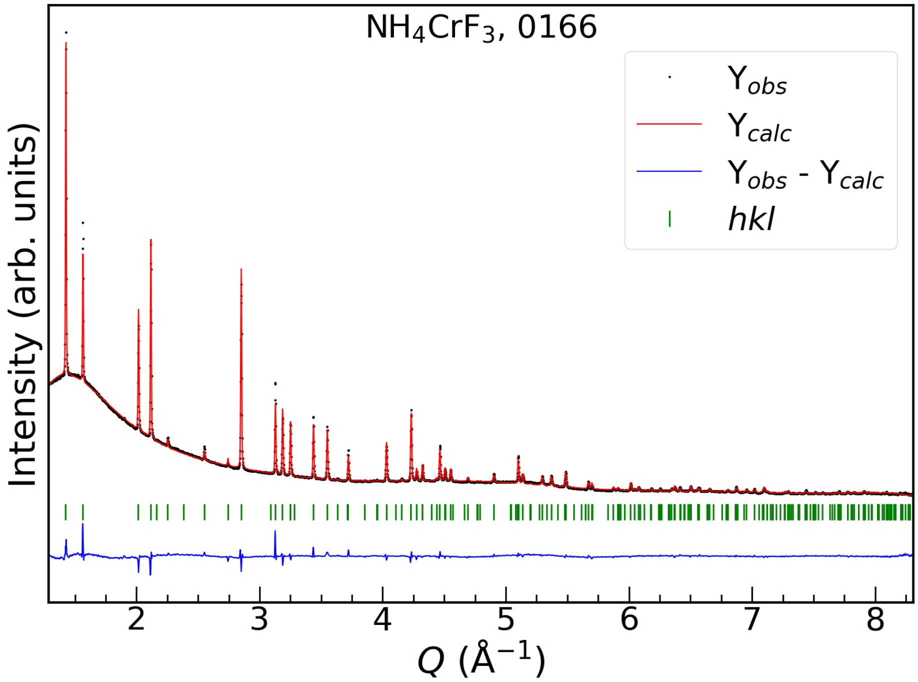

Figure S6: Rietveld refinement of NH_4CrF_3 data collected at BM31 at 500 K.

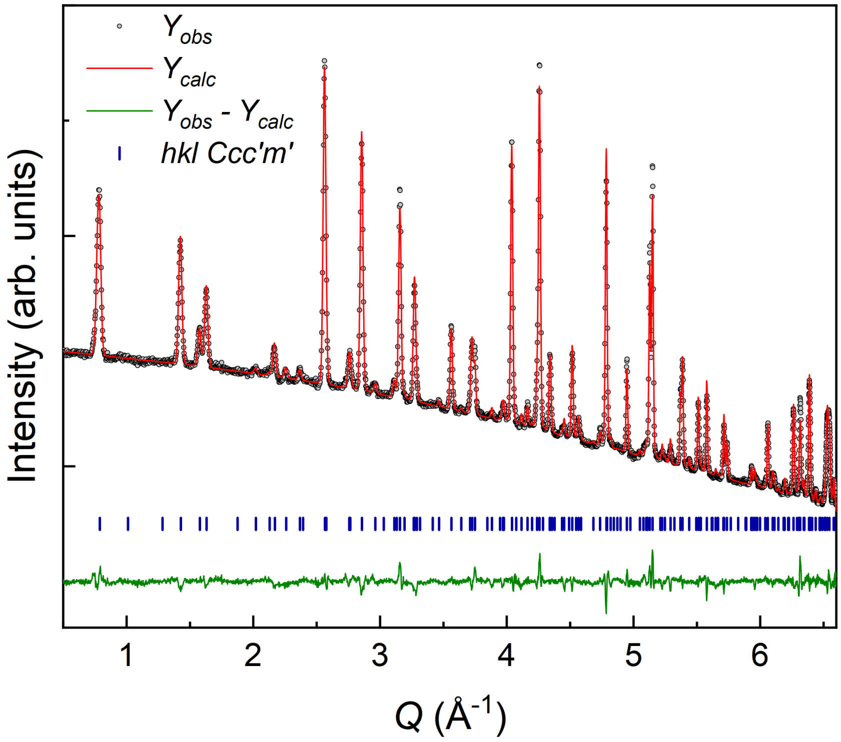

Figure S7: Rietveld refinement of the magnetic and nuclear structure of NH_4CrF_3 measured at HRPT at 1.7 K using a wavelength of 1.89 Å.

Table 1: Structural information for the magnetic structure of NH_4CrF_3 from Rietveld refinement of neutron (λ=1.89 Å) at 1.7 K. The refinement was performed in magnetic space group $Ccc'm'$ with lattice parameters of $a = b = 8.7986(2)$ Å, and $c = 7.9586(2)$ Å. The magnetic moment on chromium was refined to $M_y = 3.72(2)\ \mu_B$. M_y of the two chromium sites were restricted to have the same moment with opposite signs. M_x and M_z was restricted to zero.

Atom	x	y	z	Occ	U_{iso} (Å^2)
N1	0	0	-0.25	1	0.0023(4)
H1	-0.4062(2)	-0.0082(4)	-0.3254(2)	1	0.0231(5)
H2	-0.0082(4)	0.4062(2)	-0.8254(2)	1	0.0231(5)
Cr1	-0.25	0.25	0	1	0.0071(6)
Cr2	0.25	0.25	0.5	1	0.0071(6)
F1	-0.25	0.25	-0.25	1	0.0063(5)
F2	0.25	0.25	-0.75	1	0.0063(5)
F3	-0.52478(19)	-0.22656(17)	0	1	0.0044(4)
F4	-0.22656(17)	0.52478(19)	0.5	1	0.0044(4)

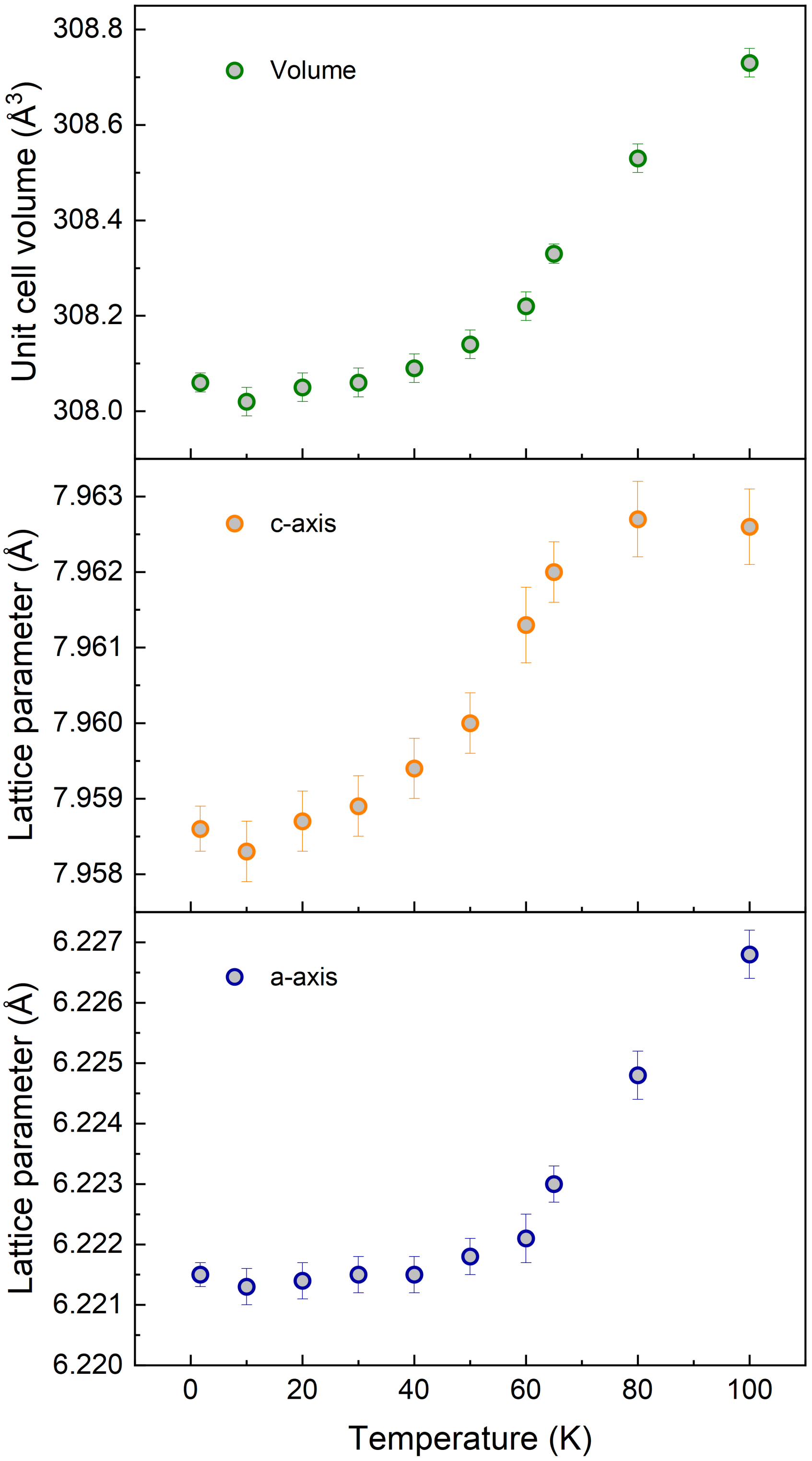

Figure S8: Lattice parameters of NH_4CrF_3 from Rietveld refinements of HRPT data.

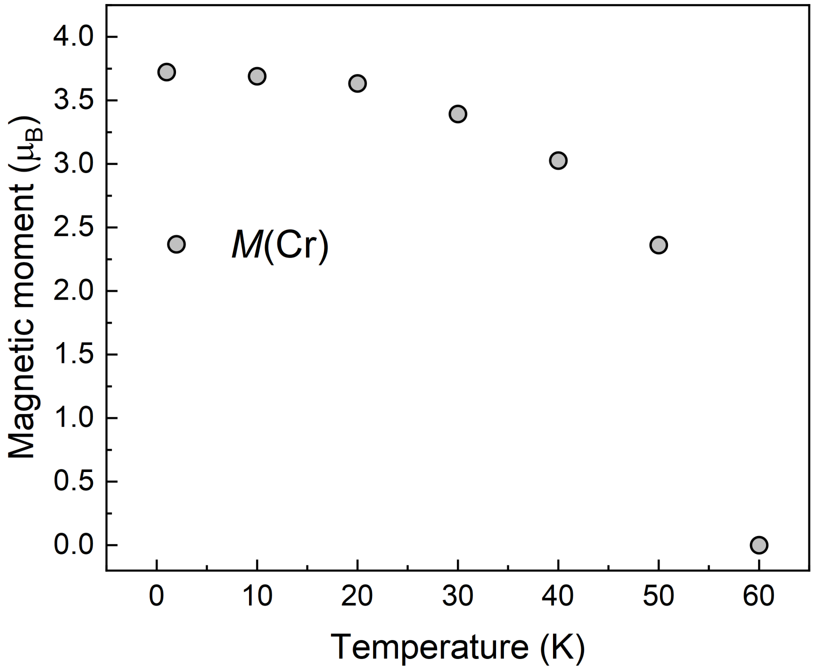

Figure S9: Refinement magnetic moment of Cr^{2+} in NH_4CrF_3 from Rietveld refinements of HRPT data.

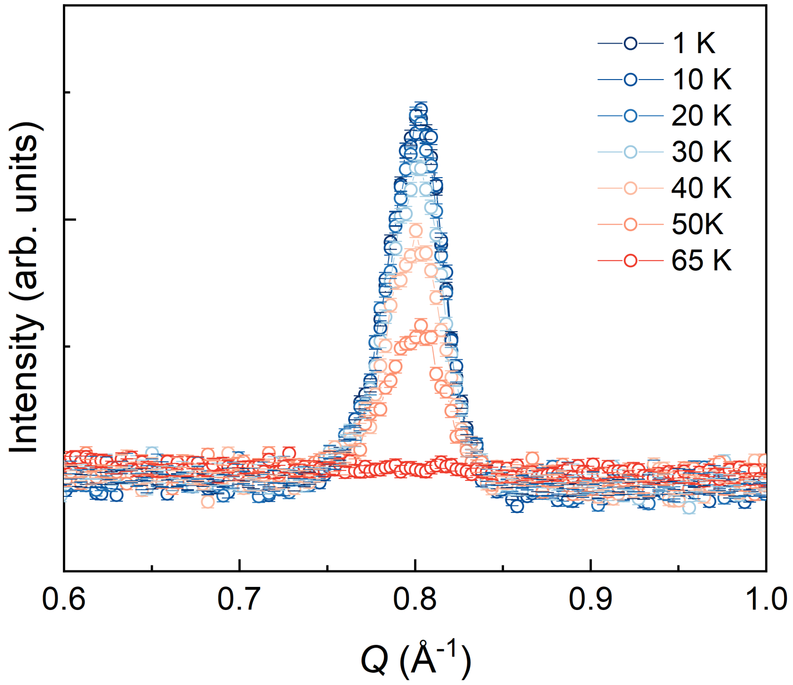

Figure S10: The intensity of the (001) reflection of NH_4CrF_3 as a function of temperature measured at HRPT.
